# Adenovirus-mediated transfer of hepatocyte growth factor gene to human dental pulp stem cells under good manufacturing practice improves their potential for periodontal regeneration in swine

**DOI:** 10.1186/s13287-015-0244-5

**Published:** 2015-12-15

**Authors:** Yu Cao, Zhenhai Liu, Yilin Xie, Jingchao Hu, Hua Wang, Zhipeng Fan, Chunmei Zhang, Jingsong Wang, Chu-Tse Wu, Songlin Wang

**Affiliations:** Molecular Laboratory for Gene Therapy & Tooth Regeneration, Beijing Key Laboratory of Tooth Regeneration and Function Reconstruction, Capital Medical University School of Stomatology, Tian Tan Xi Li No. 4, Beijing, 100050 P.R. China; Department of Stomatology, Beijing Jishuitan Hospital, No.31, Xinjiekou East Street, Xicheng District, Beijing, 100035 P.R. China; Department of Experimental Hematology, Beijing Institute of Radiation Medicine, 27 Taiping Road, Beijing, 100850 P.R. China; Department of Biochemistry and Molecular Biology, Capital Medical University School of Basic Medical Sciences, No.10 You An Men Wai Tou Tiao,, Beijing, 100069 P.R. China

**Keywords:** dental pulp stem cells, cell injection, cell sheet, hepatocyte growth factor, periodontal regeneration

## Abstract

**Introduction:**

Periodontitis is one of the most widespread infectious diseases in humans. We previously promoted significant periodontal tissue regeneration in swine models with the transplantation of autologous periodontal ligament stem cells (PDLSCs) and PDLSC sheet. We also promoted periodontal tissue regeneration in a rat model with a local injection of allogeneic bone marrow mesenchymal stem cells. The purpose of the present study is to investigate the roles of the hepatocyte growth factor (HGF) and human dental pulp stem cells (DPSCs) in periodontal tissue regeneration in swine.

**Method:**

In the present study, we transferred an adenovirus that carried HGF gene into human DPSCs (HGF-hDPSCs) under good manufacturing practice (GMP) conditions. These cells were then transplanted into a swine model for periodontal regeneration. Twenty miniature pigs were used to generate periodontitis with bone defect of 5 mm in width, 7 mm in length, and 3 mm in depth. After 12 weeks, clinical, radiological, quantitative and histological assessment of regenerated periodontal tissues was performed to compare periodontal regeneration in swine treated with cell implantation.

**Results:**

Our study showed that injecting HGF-hDPSCs into this large animal model could significantly improve periodontal bone regeneration and soft tissue healing. A hDPSC or HGF-hDPSC sheet showed superior periodontal tissue regeneration compared to the injection of dissociated cells. However, the sheets required surgical placement; thus, they were suitable for surgically-managed periodontitis treatments. The adenovirus-mediated transfer of the HGF gene markedly decreased hDPSC apoptosis in a hypoxic environment or in serum-free medium, and it increased blood vessel regeneration.

**Conclusion:**

This study indicated that HGF-hDPSCs produced under GMP conditions significantly improved periodontal bone regeneration in swine; thus, this method represents a potential clinical application for periodontal regeneration.

**Electronic supplementary material:**

The online version of this article (doi:10.1186/s13287-015-0244-5) contains supplementary material, which is available to authorized users.

## Introduction

Periodontitis results in the destruction of tooth-supporting tissues, such as bone, periodontal ligament, and cementum [1]. To date, there is no ideal therapeutic approach for achieving optimal periodontal tissue regeneration. Recent progress in tissue engineering has shown that ex vivo expanded mesenchymal stem cells (MSCs) are suitable for regenerative medicine, because of their potential for differentiating into multiple lineages [2–6].

We previously generated a swine model of periodontitis [7]. We then demonstrated that significant periodontal tissue could be regenerated by transplanting periodontal ligament stem cells (PDLSCs) into the periodontal defects in the swine model [7–9]. However, sources of PDLSCs are limited, which greatly impedes the clinical application of this approach. Alternatively, adult dental pulp stem cells (DPSCs) are easily accessible because a large number of DPSCs are produced, and the isolation methods are noninvasive compared with other adult tissue sources. Based on their multipotential differentiation properties, autologous DPSCs have been used recently for clinical periodontal regeneration [10]. In addition, angiogenic growth factors, including hepatocyte growth factor (HGF), have been employed to induce periodontal regeneration [11]. HGF was originally identified as a potent mitogen for mature hepatocytes [12]. Later, it was shown that HGF could stimulate mitogenic, morphogenic, and antiapoptotic activities in a wide variety of cells, including most epithelial and endothelial cells. Of note, HGF enhanced the regeneration of several organs, including the heart, liver, kidney, and lung [13, 14].

In this study, we cultured human dental pulp stem cells (hDPSCs), and modified them by introducing an adenovirus vector that harbored the HGF gene (Ad-HGF), under good manufacturing practice (GMP) conditions. We then transplanted these HGF gene-modified hDPSCs (HGF-hDPSCs), by cell injection or by cell sheet transplantation, into miniature pigs for periodontal regeneration. With this approach, we evaluated the roles of HGF and DPSCs in periodontal tissue regeneration. This preclinical study showed that, when DPSCs modified with Ad-HGF were injected into this large animal model, they could significantly improve periodontal bone regeneration.

## Methods

### hDPSCs cultured under GMP

Normal human impacted third molars were collected from male adults (19–29 years old) at the Dental Clinic of Beijing Stomatological Hospital under approved guidelines set by the Research Ethics Committee of Capital Medical University, China. All patients gave their written informed consent to participate. Tooth surfaces were cleaned and cut around the cementum–enamel junction (CEJ) with sterilized dental fissure burs to reveal the pulp chamber. The pulp tissue was gently separated from the crown and root, and then digested in collagenase for 1 hour at 37 °C. Single cell suspensions were obtained by passing the cells through a 70 μm strainer (Falcon; BD Labware, Franklin Lakes, NJ, USA). The hDPSCs were cultured in a GMP-compliant facility according to ISO 8 clean room standards. Briefly, our in-house qualification method involves cleaning and decontamination for facilities such as water baths, incubators, refrigerators, freezers, and centrifuges. Temperature and gases are also continuously monitored in the equipment through a wireless network system operated by contract vendors. The equipment in the facility is monitored on a daily basis. External certified vendors calibrate the biosafety cabinets and monitor air as per ISO standards and perform a viable particle count in clean rooms and the biosafety cabinet. Periodic calibrations are done to ensure functioning of the equipment as stipulated. External vendors also perform calibration and qualification of pipettes. Testing of adventitious agents is done by clinically certified laboratories. The facility was equipped with class II and class III biosafety cabinets and all other standard tissue culture equipment. We used xenobiotic-free cell culture reagents for culturing cells, as follows: animal origin-free collagenase (Worthington Biochemical Corporation, Lakewood, NJ, USA), CELLstart, EZPassage Tool, Ca/Mg-free HBSS, D/F12, TrypLE, xeno-free B27, N_2_ supplement, MSCGM-CD, and ProFreeze CDM (Invitrogen/Gibco, Carlsbad, CA, USA), human serum (Innovative Research, Inc., Novi, MI, USA), basic fibroblast growth factor-2 (bFGF-2; Peprotech, Rocky Hill, NJ, USA), TeSR2, which includes high levels of bFGF and transforming growth factor beta (TGF-β; Stem Cell Technologies, Vancouver, BC, Canada), and Nutristem Stemedia (Stemgent, San Diego, CA, USA). Nutristem Stemedia consisted of human recombinant insulin, human serum albumin, transferrin, human fibroblast growth factor, and TGF-β.

To identify putative stem cells, single cell suspensions (1 × 10^5^ cells) were seeded onto 10 cm culture dishes (Corning Costar, Cambridge, MA, USA) in alpha-modified Eagle’s medium (a-MEM; Gibco, Invitrogen), supplemented with xenobiotic-free cell culture reagents; cells were then incubated at 37 °C in 5 % carbon dioxide. Colony-forming efficiency was assessed on day 14. Aggregates of 50 cells or more were scored as colonies. All cells in this study were used after three or four passages. For each experiment, all hDPSCs were used after the same number of passages. MSC characterizations, including surface molecule expression profiles and multilineage differentiation, were performed as described previously [7].

### Adenoviral vectors and gene transfection

We constructed replication-deficient recombinant adenovirus vectors that carried the green fluorescent protein (Ad-GFP; control) or human HGF (Ad-HGF) [14]. All adenovirus vectors were constructed with the AdEasy system (Stratagene, La Jolla, CA, USA). Briefly, the human HGF gene or GFP gene was amplified by PCR and inserted into the pShuttle-CMV (Stratagene, LaJolla, CA, USA), obtaining the pShuttle plasmid pShuttle-HGF or pShuttle-GFP. pShuttle-HGF and pShuttle-GFP were then used to generate Ad-HGF and Ad-GFP, respectively, according to the manual accompanying the AdEasy system. All adenovirus vectors were purified by double CsCl density gradient ultracentrifugation, dissolved in storage buffer (Hanks’ buffer, 10 % glycerol), and stored at –80 °C. Viral particle (VP) numbers and infectious titers (infectious units/ml) were determined as described previously; and the multiplicity of infection was calculated from infectious titers. The final number of plaque-forming units was determined by titration on human embryonic kidney 293 cells under an agarose overlay.

The hDPSCs were infected with the adenovirus vectors at different multiples of infection (MOIs), in the 50–400 range. The transfection efficiency was assessed with GFP as the reporter molecule, and the infection efficiency was determined by flow cytometry.

### Enzyme-linked immunosorbent assay for HGF expression

HGF-hDPSCs were assayed for HGF expression by enzyme-linked immunosorbent assay (ELISA). Briefly, cell supernatants were collected at different time points (up to 14 days) after transfection. ELISA was conducted with a primary mouse monoclonal antibody against human HGF, and a secondary horseradish peroxidase-labeled goat antimouse immunoglobulin. Detection was performed with a spectrophotometric *o*-phenylenediamine color-developing system. The optical density was measured at 405 nm with a Microplate Manager 450 (Bio-Rad, Hercules, California, USA).

### Determination of apoptotic cells

hDPSC apoptosis was evaluated with the Annexin-V-FLUOS apoptosis staining kit (Roche Diagnostic, Rotkreuz, Switzerland). A total of 10^6^ cells were washed with phosphate-buffered saline (PBS; Invitrogen) and centrifuged at 200 × *g* for 5 minutes. The cell pellet was resuspended in 100 ml Annexin-V-FLUOS labeling solution, incubated, and analyzed with flow cytometry.

### Measurement of capillary density in periodontal soft tissues

Four miniature pigs were used to assess the effect of HGF-hDPSCs on the vascularization in periodontal tissues. The present study was approved by the animal care and use committee of Capital Medical University (reference number: AEEI-2015-089). The animal care and experimental procedures were carried out in accordance with guidelines of the Beijing Experimental Animal Management Ordinance. Approximately 1 × 10^7^ hDPSCs in 0.6 ml of 0.9 % NaCl were injected at three sites (approximately 0.2 ml per site): the mesial side, the distal side, and the middle side of the buccal gingival tissues of one mandible first molar of four pigs. The same dose of HGF-hDPSCs was injected using the same procedure into the other mandible first molar of four pigs. At 4 weeks post injection, we located the alveolar bone crest by bone sounding, and we determined its biological width. Connective tissue samples were then removed from the gingival margin to the top of the alveolar bone crest. Subsequently, all samples were sectioned in the bucco-lingual direction and then stained with hematoxylin and eosin (H & E). In each sample, five regions of interest (200× objective) were randomly imaged. The capillary vessels count was performed in these five randomly selected high-power (200× objective) microscopic fields (HPFs). The number of capillary vessels was counted by two distinct observers. As an internal control (intraobserver variation), the capillary vessels count for all samples was repeated by the same observer (YC). For external control (interobserver variation), all samples were recounted by a second independent, blinded observer (CZ). All investigators were blinded to the status of the animal. The five values were averaged to determine the number of capillary vessels per field for each sample.

### Flow cytometric analysis

To examine stem cell surface molecule expression in hDPSCs, 2.5 × 10^5^ third-passage cells were placed in 1.5 ml eppendorf tubes and fixed with 4 % paraformaldehyde for 15 minutes. Primary anti-STRO-1 and anti-CD146 antibodies were added to the tubes and incubated at room temperature for 1 hour. Next, fluorescein isothiocyanate (FITC)-conjugated or phycoerythrin (PE)-conjugated secondary antibodies were added, and the mixtures were incubated at room temperature in the dark for 45 minutes. The percentages of cells positively stained with STRO-1 and CD146 were assessed with fluorescence-activated cell sorting, in a FACSCalibur flow cytometer (Becton Dickinson Immunocytometry Systems, San Jose, California,USA).

### Carboxyfluorescein diacetate succinimidyl ester staining

Cell-cycle length was analyzed with carboxyfluorescein diacetate succinimidyl ester (CFSE) staining. The cells were harvested, washed with PBS + 0.1 % bovine serum albumin (BSA), and then cold absolute ethanol was added during vortexing of the cells. The cells were suspended in PBS + 0.1 % BSA and stained for 10 minutes at 4 °C with 5 μM CFSE (b21888; Sigma, St.Louis., MO, USA ). The staining was quenched by adding 4 volumes of ice-cold culture medium + 1 % BSA (5 minutes), and the cells were then washed twice with PBS at 4 °C. The stained cells were reintroduced into culture for 24 or 48 hours. The cells were then harvested, and the intensity of CFSE staining was assessed by fluorescence microscopy. The number of cells stained was quantified by flow cytometry with a FACScan (Becton-Dickinson, Heidelberg, Germany) and the results were analyzed with Flowing software (Version 2.5, Joseph Trotter, Scripps Research Institute, La Jolla, CA, USA).

### Multilineage differentiation potential

hDPSCs were cultured in adipogenic differentiation media and in osteogenic differentiation media. Oil Red O stain and Alizarin Red stain, respectively, were used to determine hDPSC differentiation potentials.

### Ectopic mineralized tissue formation analysis

The present study was approved by the Animal Care and Use Committee of Capital Medical University. The animal care and experimental procedures were carried out in accordance with the guidelines of the Beijing Experimental Animal Management Ordinance. In the control group, hydroxyapatite/tricalcium phosphate (HA-TCP) was transplanted subcutaneously into the dorsal region of immunodeficient female mice (BALB/c Nude, 6–8 weeks old). In the two experimental groups (*n* = 6), complexes of HA-TCP and hDPSCs or complexes of HA-TCP and HGF-hDPSCs were transplanted with the same procedure. The mice were allowed to recover for 12 weeks, and were then sacrificed for analysis.

The dissected tissues were fixed in 4 % formalin for 24 hours, decalcified with buffered 10 % ethylenediaminetetraacetic acid (EDTA, pH 8.0), and embedded in paraffin. The paraffin blocks were sectioned at a thickness of 5 μm and stained with H & E. To quantify bone formation, the area of mineralized tissue was assessed in three consecutive slices with the Image Pro 5.0 system (Media Cybernetics, Rockville, MD, USA). The bone formation area was expressed as the percentage of bone tissue in each section. The mean value of three measurements was calculated for each implant, and this value was used to calculate the mean percentage of bone formation observed in each group.

### Generation of the periodontitis model

Twenty inbred male Wuzhishan miniature pigs, aged 12 months (sexual maturity stage), body weights of 30−40 kg, were obtained from the Institute of Animal Science at the Chinese Agriculture University (Beijing, China). All surgical procedures were performed under general anesthesia, achieved with a combination of 6 mg/kg ketamine chloride and 0.6 mg/kg xylazine (intramuscular injection). After each procedure, animals were moved to a climate-controlled and light-controlled environment and allowed free access to food (liquid food) and water.

We generated a total of 40 periodontitis lesions in the first molars, as described previously [8]. After clinical assessment, a mucoperiosteal flap was raised and alveolar bone was removed using a surgical bur to create experimental periodontal bone defects in the mesial region of the maxilla and mandibular first molars. The alveolar bone defect was 5 mm in width, 7 mm in length, and 3 mm in depth, and notch-shaped marks were made on the root surface at the CEJ and the level of the floor of the defect. Three walls of the bone defect were alveolar bone, and the root surface in the bone defect was instrumented using Gracey curettes (Shanghai Kangqiao Dental Instruments Factory, Shanghai, China) to remove all periodontal ligaments as well as cementum to expose the dentin surface between two notch-shaped marks.

After creating these defects, the 20 miniature swine (two defects each) were randomly assigned to five treatment groups, with four miniature swine per group (eight defects per group). The control group received no treatment. The hDPSC injection group was injected with approximately 1 × 10^7^ hDPSCs in 0.6 ml of 0.9 % NaCl at three sites (approximately 0.2 ml per site): the mesial side of the bone defect, the distal side of the bone defect, and the middle of the bone defect. The shape of the bone defect was detected by periodontal probe (bone sounding). The tip of the needle was stopped at the bottom of the bone defect beneath the periosteum. hDPSCs were injected into the bottom of the periodontal bone defects beneath the periosteum. The HGF-hDPSC injection group received an injection of approximately 1 × 10^7^ HGF-hDPSCs with the same procedure as that described for the hDPSC injection group. Groups 4 and 5 received transplantations of hDPSC sheets and HGF-hDPSC sheets, respectively. These sheets were prepared for tissue regeneration in vivo as described in our previous report [9]. Briefly, 1.0 × 10^5^ hDPSCs or HGF-hDPSCs were cultured in 60 mm dishes with 20.0 μg/ml vitamin C for 10–15 days until they formed a sheet; each sheet contained approximately 1 × 10^7^ hDPSCs or HGF-DPSCs. One sheet was used for each transplantation. All sheets were transplanted with flap surgery. At 12 weeks after transplantation, all animals were sacrificed. Tissue samples were harvested, fixed with 4 % paraformaldehyde (Sigma-Aldrich Corp., St.Louis., MO, USA ), and assessed histologically.

### Clinical and radiological evaluations

Clinical assessments, including the probing depth (PD) and attachment loss (AL), were performed on all experimental teeth just before transplantation (week 0) and post transplantation at week 12. The PD values were established with a Williams periodontal probe (Shanghai Kangqiao Dental Instruments Factory).

Bone regeneration was examined by computed tomography (CT) (Siemens, Erlangen, Germany). CT was performed pre transplantation (week 0) and at 12 weeks post transplantation. The CT scanning conditions were 120 kV, 250 mA, a 0.75 mm slice thickness, and a 3-second slice acquisition time. Data were stored in the standard Dicom 3.0 format. Three-dimensional CT images were reconstructed to assess tissue regeneration. Images in the default Dicom format were introduced into Mimics software (Mimics, Materialise NV, Leuven, Belgium). Threshold values were set according to the Bone (CT) Scale in Mimics. Three-dimensional models were reconstructed with Optimal, a setting in Mimics. An ASCII stereolithography (STL) file of the bone was imported into Geomagic Studio (​Geomagic, Morrisville, NC,USA).

### Quantitative and histological assessments of regenerated periodontal tissues

At 12 weeks after transplantation, all animals were sacrificed and samples from the experimental areas were harvested and fixed with 4 % formaldehyde. The heights of new bone regeneration were measured with a Williams periodontal probe: the distance from the top of newly formed bone to the notch-shaped mark in the CEJ made during the operation. Each sample was measured at three different positions between the buccal and lingual sides. The mean of these three values was recorded, and the height of new bone regeneration was calculated as 7 mm minus this mean value [7]. To enable quantitative comparisons among three groups, the proportion of bone volume that occupied the virtual space of the defect was determined with Simplant® software (Dentsply Implants, Waldham, MA, USA).

Next, the harvested samples were assessed histologically. Samples that had been fixed were subsequently decalcified with buffered 10 % EDTA (pH 8.0) and embedded in paraffin. For histopathological assessments, sections (5 μm) of the bone defect region were cut in the buccal–lingual direction. All sections were then deparaffinized and stained with H & E. For histomorphometric analysis, central cuts of the samples were used for assessing bone formation; sections near the lateral limits of the defect were abandoned. Single images disclosing the center part of the defect area were superimposed to an overview image using Adobe Photoshop (Adobe Photoshop CS6; Adobe, Dublin, Ireland). To quantify bone formation, the extent of bone present in each section was evaluated semiquantitatively with histomorphometric techniques, as described previously [8]. In each group, 10 representative areas were evaluated at 5× magnification by a responsible researcher (CZ) blinded to the group assignment of the specimen. The area of bone formation was expressed as the percentage of bone present in the periodontium of the sections.

### Statistical analysis

All statistical calculations were performed with SPSS13 statistical software (SPSS, Chicago, IL, USA). Quantitative data were expressed as the mean ± standard deviation (SD). Statistical significance was determined by the independent sample test or analysis of variance. *P* ≤0.01 was considered significant.

## Results

### Intrinsic features of hDPSCs transfected with Ad-HGF in GMP conditions

**Fig. 1:**
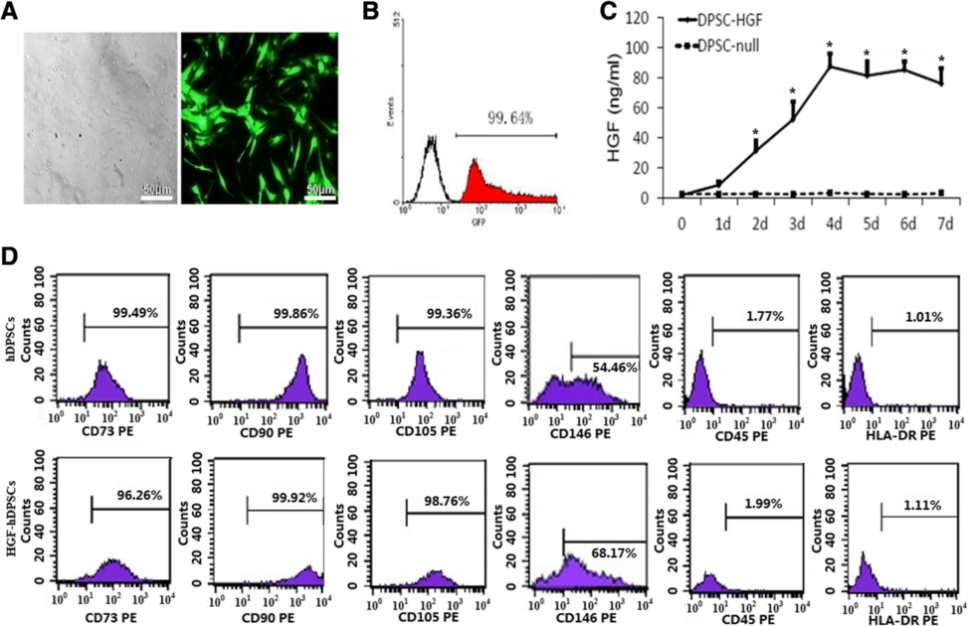
Characterization of hDPSCs cultured under GMP conditions. **a** Microscopic image (*left*) and fluorescence microscopic image (*right*) of MSCs transfected with Ad-GFP at a MOI of 150 for 48 hours. **b** Transfection efficiency of MSCs transfected with Ad-GFP. Cells were transfected at different MOI levels and cultured for 48 hours. Transfection efficiency was determined by flow cytometry. **c** Adenovirus-mediated expression of HGF in hDPSCs infected with Ad-HGF at an MOI of 150. Conditioned medium was collected at different time points, and HGF content was determined by ELISA. *P <0.05 for hDPSCs infected with Ad-HGF compared with Ad-GFP control. **d** Flow cytometric analysis of hDPSCs and HGF-hDPSCs shows expression of cell markers CD73, CD105, CD90, and CD146, and negative results for HLA-DR and CD45. *d* days, *GFP* green fluorescence protein, *hDPSC* human dental pulp stem cell, *HGF* hepatocyte growth factor, *PE* phycoerythrin

**Fig. 2:**
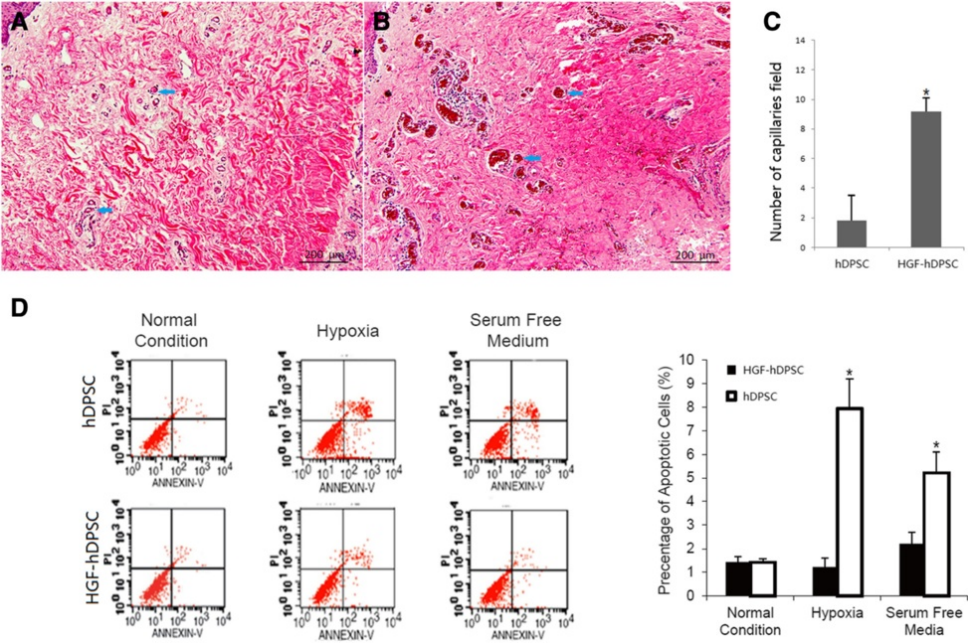
hDPSCs that overexpressed HGF prevented apoptosis and promoted neovascularization in vivo. **a**, **b** Representative photomicrographs of histological sections of periodontal soft tissues from **a** the hDPSC injection group and **b** the HGF-hDPSC injection group. *Blue arrows*, location of capillaries. **c** The number of capillaries in ischemic periodontal soft tissues was significantly higher in the HGF-hDPSC injection group compared with the hDPSC group (*P <0.05). **d** Annexin V staining for apoptosis showed that more apoptotic cells were found in the hDPSC injection group than in the HGF-hDPSC injection group (*P <0.05). *hDPSC* human dental pulp stem cell, *HGF* hepatocyte growth factor

The cells isolated from dental pulp tissue and grown into colonies under GMP conditions exhibited a typical fibroblast-like morphology (Additional file 1: Figure S1A, B). The infection efficiency of Ad-HGF reached 99.64 % in hDPSCs when the MOI was 150:1 (Fig. 1a, b). We determined whether the cultured MSCs transfected with Ad-HGF or Ad-GFP could release soluble HGF protein by assaying conditioned medium with the ELISA method. We detected a peak of 88 ng/ml HGF in HGF-hDPSC medium on day 4 post transfection (Fig. 1c). Flow cytometry analysis indicated that HGF-hDPSCs were uniformly positive for STRO-1, CD73, CD90, CD105, and CD146; additionally, they were negative for CD31, CD45, and HLA-DR (Fig. 1d). This profile was similar to that of their Ad-GFP-transfected counterparts; thus, the phenotypic profile of transfected cells was unaffected by HGF expression. Moreover, CFSE staining indicated that the proliferation efficiency of hDPSCs was not altered by HGF transduction (Additional file 1: Figure S1C). In vitro differentiation assays demonstrated that HGF-hDPSCs possessed osteogenic and adipogenic capabilities, as expected (Additional file 1: Figure S1D, E). When tissues transplanted with normal hDPSCs (Fig. 2a) were compared with tissues transplanted with HGF-hDPSCs (Fig. 2b), significantly more capillaries were observed in the tissues transplanted with HGF-hDPSCs (Fig. 2c). Moreover, when cells were exposed to a hypoxic environment or to serum-free media, apoptosis was observed in a markedly lower percentage of HGF-hDPSCs than hDPSCs (Fig. 2d).

### Ectopic mineralized tissue formation analysis

**Fig. 3:**
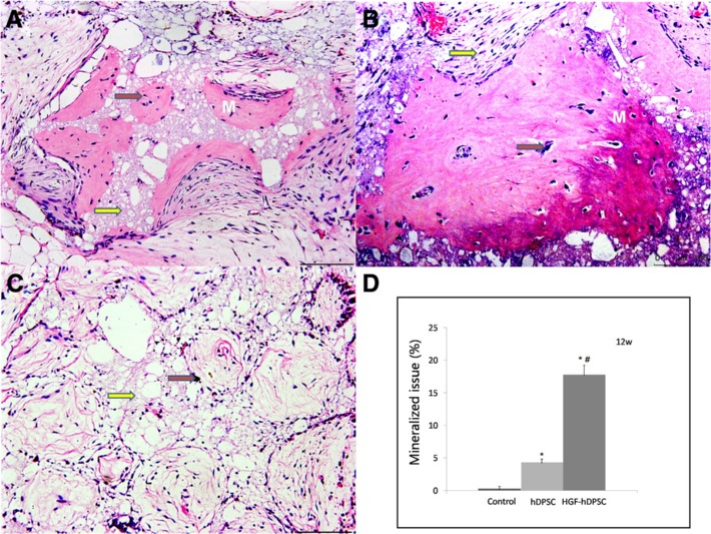
Ectopic mineralized tissue formation analysis. **a**–**c** Mineralized tissue formation was observed in both the hDPSC **a** and HGF-hDPSC **b** groups compared with control **c**, but more was observed in the HGF-hDPSC group **b. d** Compared with the control group (HA-TCP alone), more mineral tissue was formed in both the hDPSC and HGF-hDPSC groups (*P <0.01), but more was formed in the HGF-hDPSC group than in the hDPSC group (^#^ *P* <0.01). *Red arrow*, HA-TCP; *yellow arrow*, collagen fiber. Scale bar = l00 μm. *12w* 12 weeks, *hDPSC* human dental pulp stem cell, *HGF* hepatocyte growth factor, *M* mineralized tissue

At week 12 after transplantation, mineralized tissue combined with collagen fiber was more abundant in the hDPSC group (Fig. 3a) and the HGF-hDPSC group (Fig. 3b) compared with the control group (Fig. 3c). The highest amount of mineralized tissue had formed in the HGF-hDPSC group (Fig. 3b, d).

### hDPSC-mediated periodontal tissue regeneration in swine

**Fig. 4:**
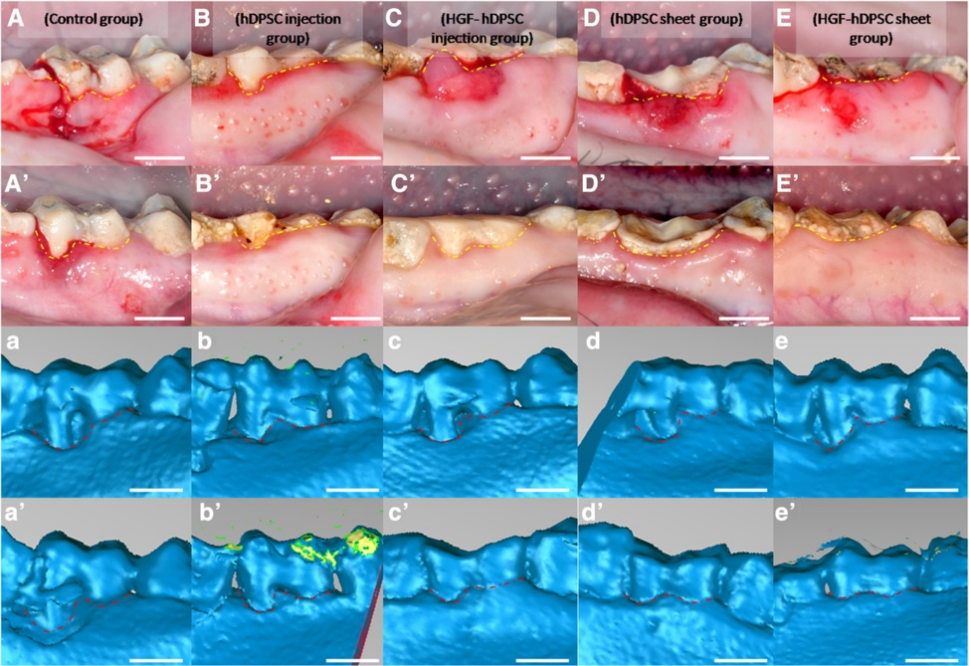
Regeneration of tissue in periodontal defects mediated by transplanted hDPSCs. Intraoral photographs were acquired *A–E* before transplantation or *A′–E′* 12 weeks after transplantation. *A′* Only limited periodontal tissues were regenerated in the control group (*yellow dotted li*ne). Marked periodontal tissue formation was found in *B′* the hDPSC injection group and *C′* the HGF-hDPSC injection group, but they did not restore tissues to healthy levels (*yellow dotted line*). Periodontal tissue regeneration mediated by *D′* the hDPSC sheet and *E′* the HGF-hDPSC sheet achieved close to normal tissue levels (*yellow dotted line*). Three-dimensional CT images were acquired *a–e* before transplantation and *a′–e′* 12 weeks after transplantation. *a′* Only limited bone regeneration was observed in the control group, but marked bone formation was observed *b′* in the hDPSC injection group, *c′* the HGF-hDPSC injection group, *d′* the hDPSC sheet group, and *e′* the HGF-hDPSC sheet group after cell transplantation (*red dotted line*). *Yellow dotted line*, gingival margin; *red dotted line*, bone margin. *hDPSC* human dental pulp stem cell, *HGF* hepatocyte growth factor

**Fig. 5:**
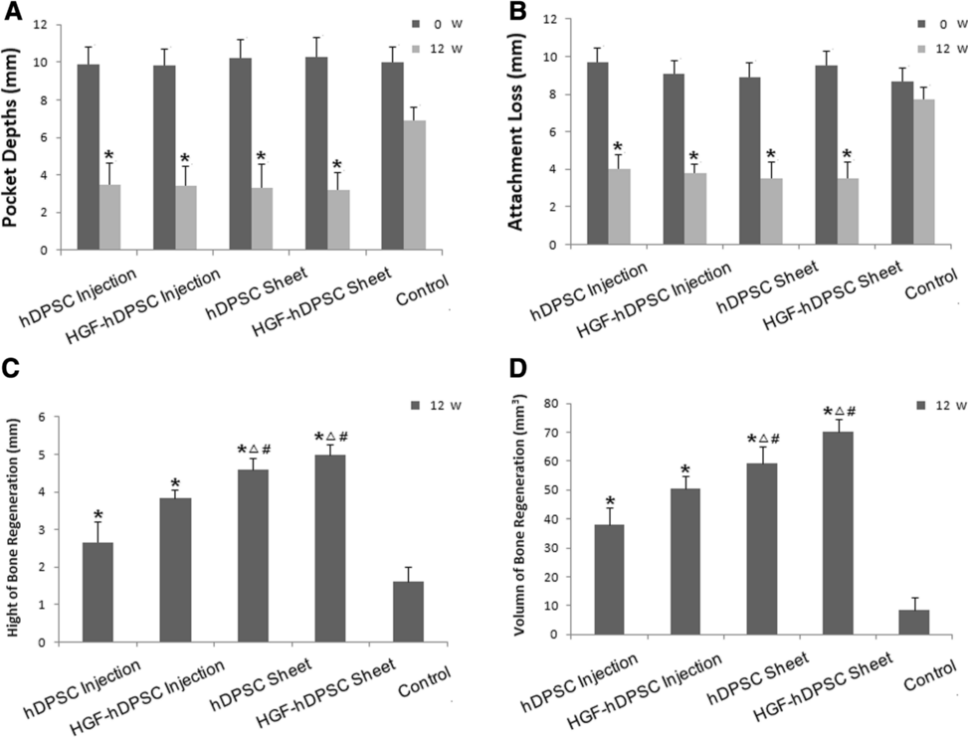
Clinical qualitative assessments of periodontal and bone tissues regenerated with dissociated cells and cellular sheet transplantations in miniature pigs. **a**, **b** Clinical assessments of the periodontal tissues. At week 0, there was no significant difference in **a** PD or **b** AL among the five groups. At 12 weeks post transplantation, the PD and AL were significantly improved in the groups that received an hDPSC injection, HGF-hDPSC injection, hDPSC sheet, or HGF-hDPSC sheet, compared with those in the control group (untreated) (*P <0.01, *n* = 8). **c** Bone regeneration height was greatest in the HGF-hDPSC sheet group, but bone regeneration heights were greater in all treatment groups compared with the control group (*P <0.01). The bone regeneration heights in the hDPSC sheet and HGF-hDPSC sheet groups were greater than those in the hDPSC injection (^Δ^ *P* <0.01) and in the HGF-hDPSC injection (^#^ *P* <0.01) groups. **d** Bone regeneration volume was largest in the HGF-hDPSC sheet group. Bone regeneration volumes in the hDPSC injection, HGF-hDPSC injection, and hDPSC sheet groups were also larger than that in the control group (*P <0.01). Bone regeneration volumes were larger in the hDPSC sheet and HGF-hDPSC sheet groups than in the hDPSC injection group (^Δ^ *P* <0.01). Bone regeneration volumes were larger in the hDPSC sheet and HGF-hDPSC sheet groups compared with the HGF-hDPSC injection group (^#^ *P* <0.01). *hDPSC* human dental pulp stem cell, *HGF* hepatocyte growth factor, *w* weeks

We generated periodontitis lesions in miniature swine, and then transplanted hDPSCs or HGF-hDPSCs into the defects. We transplanted either cell sheets or disassociated cells to compare their capacities for soft tissue healing and alveolar bone regeneration. The animals were sacrificed at 12 weeks post transplantation, and intraoral photographs were acquired. We found that at 12 weeks after transplantation only limited periodontal soft tissues were regenerated in the control group (Fig. 4A, A′). In contrast, marked periodontal tissue formation was found in the hDPSC injection group (Fig. 4B′) and the HGF-hDPSC injection group (Fig. 4C′), but the tissues were not restored to healthy levels. However, periodontal soft tissue in the hDPSC sheet group (Fig. 4D, D′) and the HGF-hDPSC sheet group (Fig. 4E, E′) was restored to close to normal levels. Three-dimensional CT images also indicated that limited bone formation occurred in the control group (Fig. 4a, a′). In contrast, marked bone regeneration occurred in the hDPSC injection group (Fig. 4b, b′), the HGF-hDPSC injection group (Fig. 4c, c′), the hDPSC sheet group (Fig. 4d, d′), and the HGF-hDPSC sheet group (Fig. 4e, e′). At this time, the AL values were 4.0 ± 0.6 mm in the hDPSC injection group, 3.8 ± 0.5 mm in the HGF-hDPSC injection group, 3.5 ± 0.7 mm in the hDPSC sheet group, 3.5 ± 1.3 mm in the HGF-hDPSC sheet group, and 7.7 ± 0.7 mm in the untreated control group (Fig. 5b). Statistical analysis indicated that both the hDPSC sheet treatment and the hDPSC injection significantly improved periodontal tissue regeneration compared with the control group (Fig. 5). The heights of newly regenerated bone were significantly higher in all treatment groups compared with the control group (Fig. 5c). Based on the CT scan and three-dimensional CT images, the regenerated alveolar bone volumes in the hDPSC injection group, the HGF-hDPSC injection group, the hDPSC sheet group, and the HGF-hDPSC sheet group were 38.2 ± 5.3 mm^3^, 47.3 ± 4.9 mm^3^, 58.8 ± 3.8 mm^3^, and 69.3 ± 3.9 mm^3^, respectively. The bone volumes in all treatment groups were significantly larger than the volume in the control group (0.5 ± 2.3 mm^3^, Fig. 5d).

**Fig. 6:**
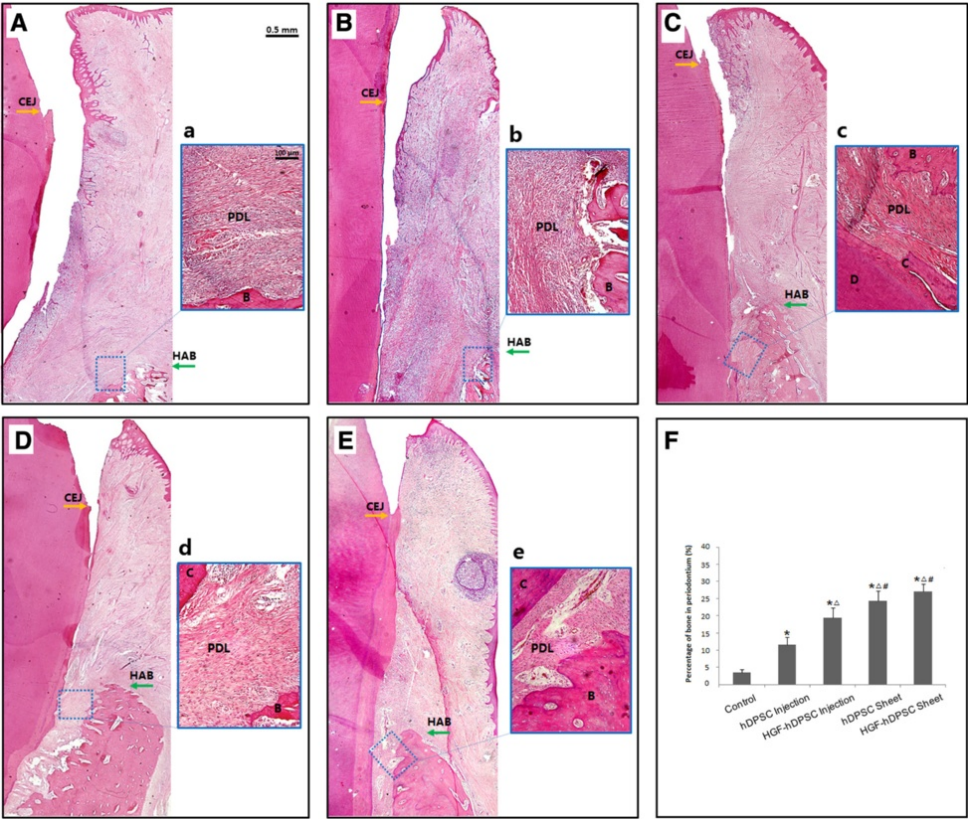
Whole-view histopathological assessments of periodontal tissue regeneration in H & E stained sections. New periodontal tissue regeneration within the periodontal defect is shown for the **A** control group, **B** hDPSC injection group, **C** HGF-hDPSC injection group, **D** hDPSC sheet group, and **E** HGF-hDPSC sheet group. New bone, cementum, and periodontal ligament were regenerated in the periodontal defect areas that received *a* no treatment, or treatments of *b* hDPSC injections, *c* HGF-hDPSC injections, *d* hDPSC sheets, or *e* HGF-hDPSC sheets. Alveolar bone regeneration in **A** the control group was much less pronounced than that observed in the **C** HGF-hDPSC injection group, **D** hDPSC sheet group, and **E** HGF-hDPSC sheet group. Newly formed Sharpey’s fibers were observed penetrating into the newly regenerated cementum in the *c* HGF-hDPSC injection group, *d* hDPSC sheet group, and *e* HGF-hDPSC sheet group. **F** The percentages of periodontal bone in the hDPSC injection, HGF-hDPSC injection, hDPSC sheet, and HGF-hDPSC sheet groups were significantly higher than that of the control group (*P <0.01). The percentages of periodontal bone were higher in the HGF-hDPSC injection, hDPSC sheet, and HGF-hDPSC sheet groups than in the hDPSC injection group (^Δ^ *P* <0.01). The percentages of periodontal bone were higher in the hDPSC sheet and HGF-hDPSC sheet groups than in the HGF-hDPSC injection group (^#^ *P* <0.01). *B* bone, *C* cementum, *CEJ*, cementum–enamel junction, *D* dentin, *HAB* height of alveolar bone, *hDPSC* human dental pulp stem cell, *HGF* hepatocyte growth factor, *PDL* periodontal ligament

The 12-week posttransplantation experimental tissue samples were sectioned in the buccal–lingual direction and stained with H & E to provide a view of the entire section. New periodontal tissue regeneration within the periodontal defects was significantly less pronounced in the control group (Fig. 6A) compared with the regeneration observed in the treatment groups (Figs. 6B–E). Both bone and periodontal ligament tissues were regenerated within the periodontal defect areas in all of the hDPSC groups (Figs. 6b–e). Alveolar bone regeneration was also more pronounced in the HGF-hDPSC injection group (Fig. 6C), the hDPSC sheet group (Fig. 6D), and the HGF-hDPSC sheet group (Fig. 6E) than in the control group (Fig. 6A, F). Newly formed Sharpey’s fibers had penetrated into the newly regenerated mineral tissues in the HGF-hDPSC injection group (Fig. 6c), the hDPSC sheet group (Fig. 6d), and the HGF-hDPSC sheet group (Fig. 6e). The percentages of periodontal bone in the hDPSC injection, HGF-hDPSC injection, hDPSC sheet, and HGF-hDPSC sheet groups were significantly higher than that of the control group (*P <0.01). The percentages of periodontal bone were higher in the HGF-hDPSC injection, hDPSC sheet, and HGF-hDPSC sheet groups than in the hDPSC injection group (^Δ^*P* <0.01). The percentages of periodontal bone were higher in the hDPSC sheet and HGF-hDPSC sheet groups than in the HGF-hDPSC injection group (^#^*P* <0.01) (Fig. 6f).

## Discussion

The present study investigated the feasibility of transplanting hDPSC/HGF-hDPSCs and hDPSC/HGF-hDPSC sheets for treating periodontitis in a large animal model. At 12 weeks after transplantation, we found that new alveolar bone had regenerated in the hDPSC/HGF-hDPSC injection groups and the hDPSC/HGF-hDPSC sheet groups. In contrast, in the control group, periodontal defects were largely filled with fibrous tissue and epithelium, and limited, irregular new attachment was observed. These results suggested that the hDPSC injections and hDPSC sheets contributed significantly to periodontal bone regeneration. Notably, the HGF-hDPSC injection exhibited more periodontal tissue regeneration capacity than the hDPSC injection. CT scan analyses showed a significantly larger volume of periodontal alveolar bone in the HGF-hDPSC injection group than in the hDPSC injection group. However, based on intraoral photographs acquired at 12 weeks post injection, the hDPSC and HGF-hDPSC injections did not restore soft tissues to healthy levels, in contrast to our findings in the hDPSC and HGF-hDPSC sheet groups. CT scan analyses also showed that the volumes of periodontal alveolar bone in the hDPSC and HGF-hDPSC sheet groups were significantly larger than those in the hDPSC and HGF-hDPSC injection groups. Periodontal soft tissues were healed to nearly normal levels in the HGF-hDPSC/hDPSC sheet group. This result suggested that the HGF-hDPSC/hDPSC sheet could be the best regenerative method of those tested.

Our research group previously generated a swine model of periodontitis. In that model, we induced significant periodontal tissue regeneration by treating with PDLSCs mixed with HA/TCP scaffolds [7], allogeneic PDLSC sheets [8], or vitamin C-treated PDLSC sheets [9]. Compared with PDLSCs, DPSCs represented a richer tissue source and were easier to isolate. It was previously reported that autologous DPSCs could be used to regenerate intrabony periodontal tissues to restore defects [10]. We hypothesized that a local injection of DPSCs might allow transplanted cells to migrate into even narrow, small recipient sites of local periodontal defects, including periodontal intrabony defects, without damaging soft tissues or creating an osteotomy. This local injection is an easy, mildly-invasive approach for regenerating lost bone, cementum, and periodontal ligament, but at the same time it can preserve soft tissue attachments, which may be particularly important when treating mild periodontitis. Cell injection therapy is a commonly used approach for treating a variety of diseases, including the regeneration of tissues after a spinal cord injury or myocardial infarction [15, 16]. Our research group previously injected a BMMSC suspension into a rat periodontitis model and demonstrated that a local injection of BMMSCs could repair tissues that were defective due to periodontitis [17]. In this study, new periodontal tissues were regenerated 12 weeks after the hDPSC/HGF-hDPSC injections.

Cell sheets [18], which were designed to avoid the shortcomings of traditional tissue engineering, have been used extensively and were shown to be beneficial for many clinical applications, such as cell transplantation and tissue regeneration. When cultured cells are harvested as intact sheets, along with their corresponding extracellular matrix (ECM), they can be easily attached to host tissues, even wound sites, with minimal cell loss. A cell sheet has a three-dimensional macrostructure that mimics the physiological functions of the ECM. Cell sheets have the advantage of eliminating the use of scaffolds; thus, they avoid strong inflammatory responses that are induced when biodegradable scaffolds are broken down. In this study, new alveolar bone and periodontal soft tissues were regenerated to nearly normal levels at 12 weeks after the administration of cell sheets. However, effective cell sheet transplanting requires open flap surgery, which may be traumatic for patients; such treatment is thus more suitable for combination with surgical periodontal treatment.

We also transplanted HGF-modified hDPSCs for treating periodontitis in miniature pigs to evaluate the role of HGF in periodontal tissue regeneration. HGF, a member of the endothelial growth factor family, has been shown to induce angiogenesis in vivo [13]. It is well known that HGF enhances the regeneration of organs, such as the liver, kidney, and lung [14]. In addition to its angiogenic activity [19–21], it has been well documented that HGF can prevent cell death. Because of its inhibitory effect on fibrosis, HGF protein delivery or HGF gene therapy approaches improved renal fibrosis and renal dysfunction [22]. HGF is also a healing factor, with cytoprotective effects in conditions associated with inflammatory diseases, including periodontitis. A number of studies demonstrated that HGF levels were elevated in patients with chronic periodontitis [23, 24]. However, despite high concentrations, HGF may appear in a form with reduced biological activity at local inflammation sites [25]. Previous studies have emphasized the importance of endogenous, biologically-active HGF in regeneration and healing during chronic inflammatory diseases. Several studies have shown that, in the cardiovascular system [26–29], endogenous HGF was present in a reduced biologically-active form. However, administration of exogenous HGF was shown to prevent tissue fibrosis and dysfunction in chronic disease models [30–32].

In the present study, because hDPSCs did not secrete sufficient angiogenic factors to induce angiogenesis in vivo, we transduced the human HGF cDNA into hDPSCs with the adenoviral system, which achieved high HGF secretion in vitro. Autocrine HGF did not suppress the differentiation potential of hDPSCs in vitro. In this study, we also found that, when HGF-hDPSCs and hDPSCs were subjected to a hypoxic environment or serum-free media, apoptosis occurred markedly less frequently in HGF-hDPSCs than in hDPSCs. This result suggested that HGF functioned as a protective factor. Our results confirmed that the HGF-hDPSC injection and HGF-hDPSC sheet promoted the growth of significantly larger volumes of periodontal alveolar bone than the hDPSC injection and untreated control. However, the biological activity of the HGF secreted by implanted hDPSCs requires further investigation. The results from the present study showed that HGF-hDPSC injection promoted the growth of significantly larger volumes of periodontal alveolar bone than the hDPSC injection, while DPSC sheets and HGF-DPSC sheets did not make a significant difference in clinical outcomes. In general, the effect of periodontal regeneration using DPSC sheets was better than that for DPSC injection. Since cell sheet transplanting requires open flap surgery, which may be traumatic for patients, DPSC injection with or without HGF transfection should be considered as an effective non-surgical therapy; DPSC sheets are an appropriate surgical therapy for periodontitis.

## Conclusions

This study supported the feasibility of using hDPSCs cultured under GMP guidelines as a potential stem cell technology for periodontal regeneration. Our data demonstrated that hDPSC/HGF-hDPSC injections and hDPSC/HGF-hDPSC sheets provided appropriate therapy for periodontitis, by inducing significant periodontal bone regeneration and soft tissue recovery.

## Additional file

Additional file 1:
**Figure S1.** Showing biological functions of hDPSCs and HGF-hDPSCs under GMP conditions. Representative phase-contrast microscopic photographs of hDPSCs after culturing for **A** 7 days (first passage) and **B** 18 days (fourth passage); the morphology is typical of fibroblast-like cells. **C** The CFSE test indicated that the proliferation index was not significantly different between the hDPSCs and HGF-hDPSCs. **D, E** The differentiation potential of hDPSCs is demonstrated by **D** staining with Oil Red O and **E** staining with Alizarin Red S. (TIF 2169 kb)

## Abbreviations

Ad-GFP: Adenovirus vector that harbored the green fluorescent protein
Ad-HGF: Adenovirus vector that harbored the HGF gene
AL: Attachment loss
bFGF-2: Basic fibroblast growth factor-2
BSA: Bovine serum albumin
CEJ: Cementum–enamel junction
CFSE: Carboxyfluorescein diacetate succinimidyl ester
CT: Computed tomography
DPSC: Dental pulp stem cell
ECM: Extracellular matrix
EDTA: Ethylenediaminetetraacetic acid
ELISA: Enzyme-linked immunosorbent assay
FITC: Fluorescein isothiocyanate
GFP: Green fluorescent protein
GMP: Good manufacturing practice
H & E: Hematoxylin and eosin
HA-TCP: Hydroxyapatite/tricalcium phosphate
hDPSC: Human dental pulp stem cell
HGF: Hepatocyte growth factor
HGF-hDPSC: HGF gene modified human dental pulp stem cell
HPF: High-power microscopic field
MOI: multiple of infection
MSC: Mesenchymal stem cell
PBS: Phosphate-buffered saline
PD: Probing depth
PDLSC: Periodontal ligament stem cell
PE: Phycoerythrin
SD: Standard deviation
STL: Stereolithography
TGF-β: Transforming growth factor beta
VP: Viral particle
α-MEM: Alpha-modified Eagle’s medium
