# Supplementary figures and images for: Adenovirus-mediated transfer of hepatocyte growth factor gene to human dental pulp stem cells under good manufacturing practice improves their potential for periodontal regeneration in swine
Source: Stem Cell Res Ther. 2015 Dec 15;6:249. doi: 10.1186/s13287-015-0244-5 (PMC4681125; doi:10.1186/s13287-015-0244-5)

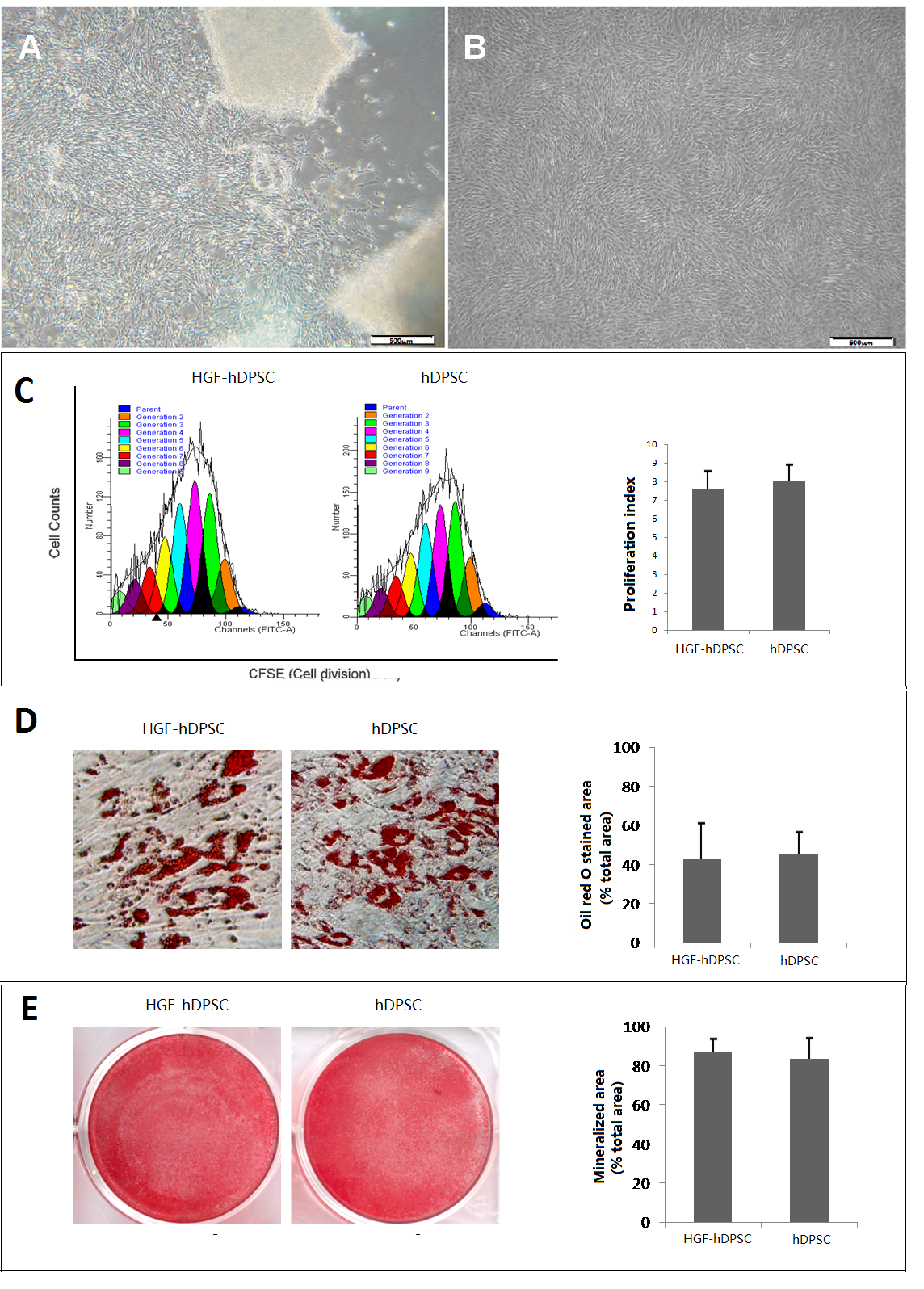

Supplement: Additional file 1: — Figure S1. Showing biological functions of hDPSCs and HGF-hDPSCs under GMP conditions. Representative phase-contrast microscopic photographs of hDPSCs after culturing for A 7 days (first passage) and B 18 days (fourth passage); the morphology is typical of fibroblast-like cells. C The CFSE test indicated that the proliferation index was not significantly different between the hDPSCs and HGF-hDPSCs. D, E The differentiation potential of hDPSCs is demonstrated by D staining with Oil Red O and E staining with Alizarin Red S. (TIF 2169 kb) [file 13287_2015_244_MOESM1_ESM.tif]
